# Supplementary material for: A whole-brain model of the neural entropy increase elicited by psychedelic drugs
Source: Sci Rep. 2023 Apr 17;13:6244. doi: 10.1038/s41598-023-32649-7 (PMC10110594; doi:10.1038/s41598-023-32649-7)
Supplement: Supplementary file 1 — Supplementary Information. [file 41598_2023_32649_MOESM1_ESM.pdf]

Supplementary Material: A mechanistic model of the neural entropy increase elicited by psychedelic drugs

Rubén Herzog[1,*], Pedro A.M. Mediano[2,3], Fernando E. Rosas[4,5,6], Paul Lodder[7], Robin Carhart-Harris[5,8], Yonatan Sanz Perl[9,10,11], Enzo Tagliazucchi[9,10,12,], and Rodrigo Cofre[13,14]**

[1]Centro Interdisciplinario de Neurociencia de Valparaíso, Universidad de Valparaíso, Pje Harrington 287, 2360103 Valparaíso, Chile.
[2]Department of Psychology, University of Cambridge, Cambridge CB2 3EB, UK
[3]Department of Computing, Imperial College London, London SW7 2DD, UK
[4]Department of Informatics, University of Sussex, Brighton BN1 9RH, UK
[5]Centre for Psychedelic Research, Department of Brain Science, Imperial College London, London SW7 2DD, UK
[6]Centre for Complexity Science, Imperial College London, London SW7 2AZ, UK
[7]Informatics Institute, University of Amsterdam, P.O. Box 94323, 1090 GH Amsterdam.
[8]Psychedelics Division, Neuroscape, University of California San Francisco, San Francisco, CA, USA.
[9]Buenos Aires Physics Institute and Physics Department, University of Buenos Aires, Buenos Aires, Argentina
[10]National Scientific and Technical Research Council, Buenos Aires, Argentina
[11]Universidad de San Andres, Buenos Aires, Argentina
[12]Latin American Brain Health Institute (BrainLat), Universidad Adolfo Ibáñez, Santiago, Chile
[13]CIMFAV-Ingemat, Facultad de Ingeniería, Universidad de Valparaíso, Valparaíso, Chile.
[14]Institute of Neuroscience (NeuroPSI), Paris-Saclay University, Centre National de la Recherche Scientifique (CNRS), Gif-sur-Yvette, France
[*]ruben.herzog@postgrado.uv.cl
[**]nztglzcch@gmail.com

ABSTRACT

Psychedelic drugs, including lysergic acid diethylamide (LSD) and other agonists of the serotonin 2A receptor (5HT2A-R), induce drastic changes in subjective experience, and provide a unique opportunity to study the neurobiological basis of consciousness. One of the most notable neurophysiological signatures of psychedelics, increased entropy in spontaneous neural activity, is thought to be of relevance to the psychedelic experience, mediating both acute alterations in consciousness and long-term effects. However, no clear mechanistic explanation for this entropy increase has been put forward so far. We sought to do this here by building upon a recent whole-brain model of serotonergic neuromodulation, to study the entropic effects of 5HT2A-R activation. Our results reproduce the overall entropy increase observed in previous experiments *in vivo*, providing the first model-based explanation for this phenomenon. We also found that entropy changes were not uniform across the brain: entropy increased in all regions, but the larger effect were localised in visuo-occipital regions. Interestingly, at the whole-brain level, this reconfiguration was not well explained by 5HT2A-R density, but related closely to the topological properties of the brain's anatomical connectivity. These results help us understand the mechanisms underlying the psychedelic state and, more generally, the pharmacological modulation of whole-brain activity.

Supplementary Subsection 1. Model fit to empirical BOLD data.

A Bayesian optimization algorithm was implemented to simultaneously optimize the global coupling parameter G, and α, a global parameter that controls the local inhibitory feedback parameter. The cost function was defined as the Kolmogorov-Smirnof distance (K-S) between the empirical and the simulated functional connectivity dynamics (FCD) distribution. Resting state BOLD signals of the placebo condition were used to fit the model associated to the non-drug condition. The data set and processing was exactly the same than the one used in Ref.[1]. The optimization stopped after 10 consecutive iterations with improvements lower than 1%. The optimal average K-S (0.198 $\pm$ 0.068) was found at G=2.4 and α 1.5 (Supplementary Figure 1A), where also the average firing rates fell within the biologically plausible[2] 3-4 Hz range (Supplementary Figure 1B,F). Once the model was fit to the placebo data, the neuromodulatory gain for the excitatory (nm_E) and the inhibitory (nm_I) population were optimized such that the K-S of the FCD between the model and the data associated to LSD BOLD signals is minimized. There was several regions where the K-S were minimized (Supplementary Figure 1D), so, for simplicity, we only used solutions were both neuromodulatory had the same magnitude, obtaining an average K-S of 0.120 $\pm$ 0.082. Then, we compared the K-S values of the models fitted to their respective data sets (Supplementary Figure 1E), finding an improvement of the goodness of fit of the model with neuromodulation to LSD data.

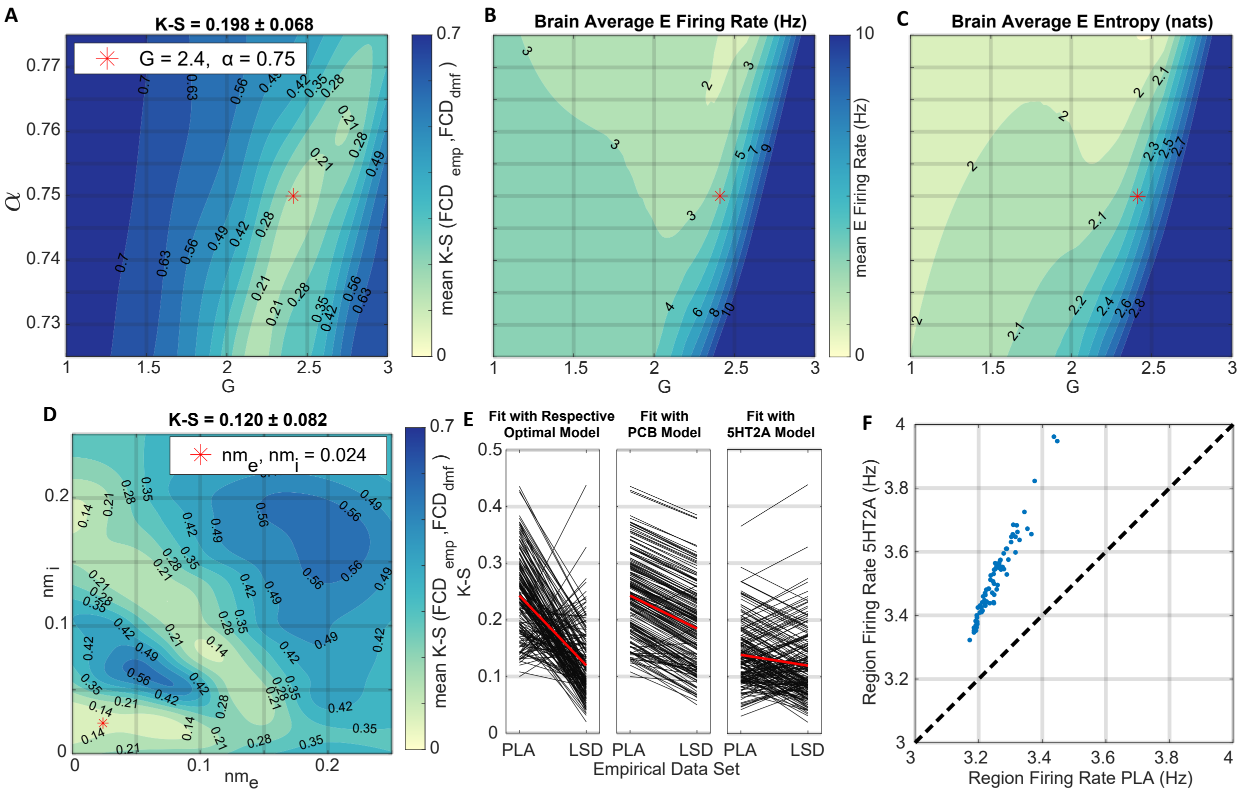

Supplementary Figure 1. Model fit to empirical BOLD data. (**A**) Estimated average K-S distance between the empirical FCD obtained from placebo resting state condition and the FCD obtained with the model. G and α were optimized by Bayesian optimization. The corresponding average excitatory firing rates, and entropies are shown in **B** and **C**, respectively. (**B,C**) Average firing rate and entropy for the excitatory pool, respectively, as a function of the two free parameters. (**D**) Estimated average K-S distance between the empirical FCD obtained from LSD resting state condition and the FCD obtained with the model with neuromodulation (5HT2A). nm_e and nm_i were optimized by Bayesian optimization. (**E**) Goodness of fit of models to their corresponding data sets, and cross-goodness of fit to data, i.e. using the placebo model for fitting LSD and the LSD model to fit placebo. The model with neuromodulation improves the goodness of fit to empirical LSD data, respect to the other comparisons. (**F**) Scatter plots of the excitatory firing rates in both simulated conditions.

Supplementary Subsection 2. Heterogeneous entropy changes induced by 5HT2A-R activation on anatomical and functional groupings of brain regions.

We asked whether the heterogeneous changes of entropy induced by 5HT2A-R activation could be explained by grouping the AAL brain regions according to anatomical and functional criteria. Regarding anatomical criteria brain regions can be

spatially split into 8 major non-overlapping anatomical groups: Frontal, Temporal, Parietal, Occipital, Limbic, Sensorimotor, Cingulate and Subcortical.[3] Regarding the functional grouping, we used the Resting State Networks, a functional grouping of brain regions based on the observed spatio-temporal patterns of BOLD signals during resting state activity.[4] These groups are Salience (Sal), Fronto-Parietal (FPN), Default Mode (DMN), Primary Visual (Vis), Extrastiate Cortex (EC), Auditory (Aud), Sensorimotor (SM), and Executive Control (EC). The first one, Sal, was obtained from Lee*et al.*[5], the second and third, FPN and DMN, were obtained from Oliver *et al.*[6], and the rest from Beckmann *et al.*[4] Brain regions can potentially belong to different functional groups.

The effect of 5HT2A-R activation on entropy is heterogeneous also at the level of anatomical groups (Supplementary Figure 2A). However, Occipital and Cingulate regions show a strong tendency to increase their entropy, in agreement with entropy increases in these regions observed in human experiments with serotonergic psychedelics.[7,8] Regarding the functional grouping (Supplementary Figure 2B), we also found that the effect of 5HT2A-R activation on regional entropy (Fig. 3B) is heterogeneous within groups. In particular, in the Vis networks showed the larger tendency to increase.

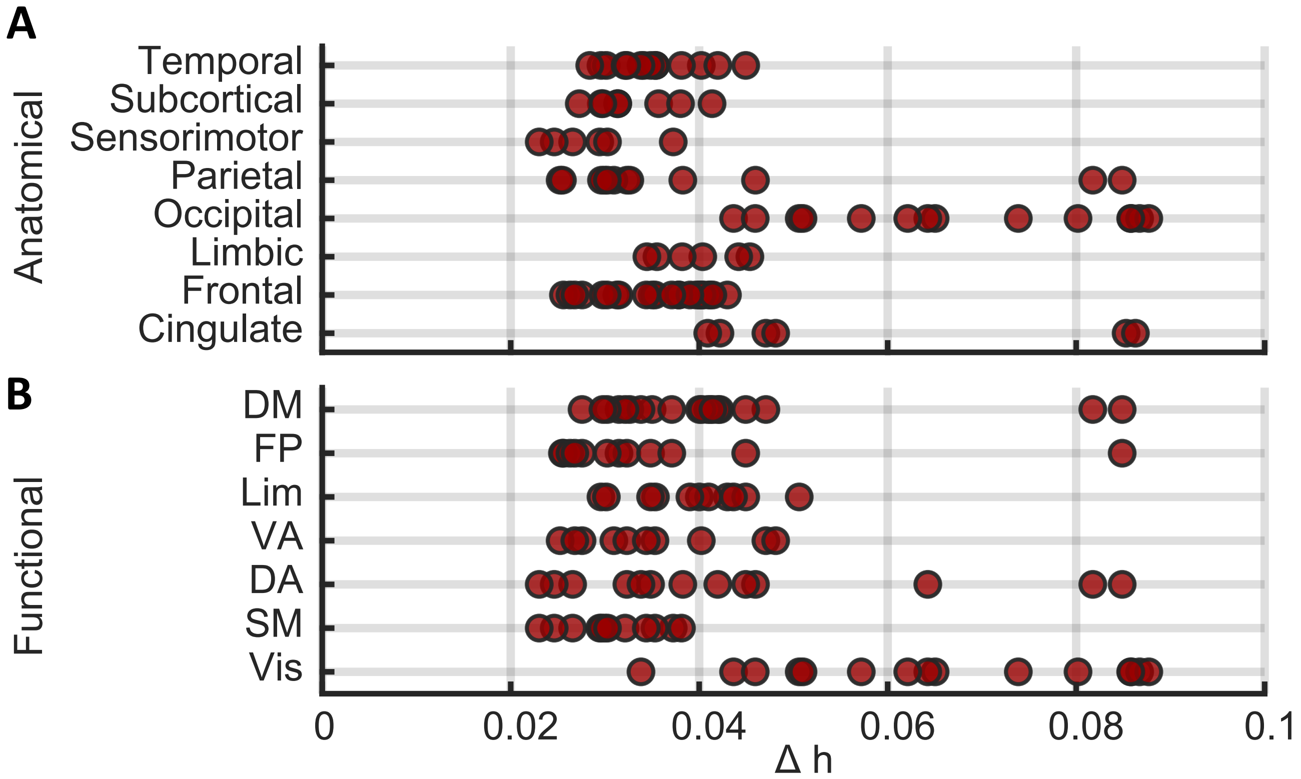

Supplementary Figure 2. The effect of 5HT2A-R activation is not explained by anatomical or functional groupings. (**A**) 5HT2A-R activation heterogeneously change entropy in regions belonging to the same anatomical group. (**B**) Entropy also changed in a heterogeneous way when functional grouping is considered. Note the special case of Vis network, which included region with larger increase.. Circles are averages computed from 1000 simulations.

Supplementary Subsection 3. Connectivity strength is the best predictor for entropy changes among local connectivity measures.

We control the role of local connectivity on the observed entropy changes (Δh_n) induced by 5HT2A-R activation using as predictors for Δh_n other local connectivity measures as: degree,[9] eigenvector centrality,[9] communicability,[10] page-rank,[9], closeness centrality,[11], and s-core[12]. Despite communicability exhibits a very similar pattern to strength, the latter has a simpler interpretation than the former. Thus, we confirmed that local connectivity strength is the best linear predictor of entropy changes among other centrality measures.

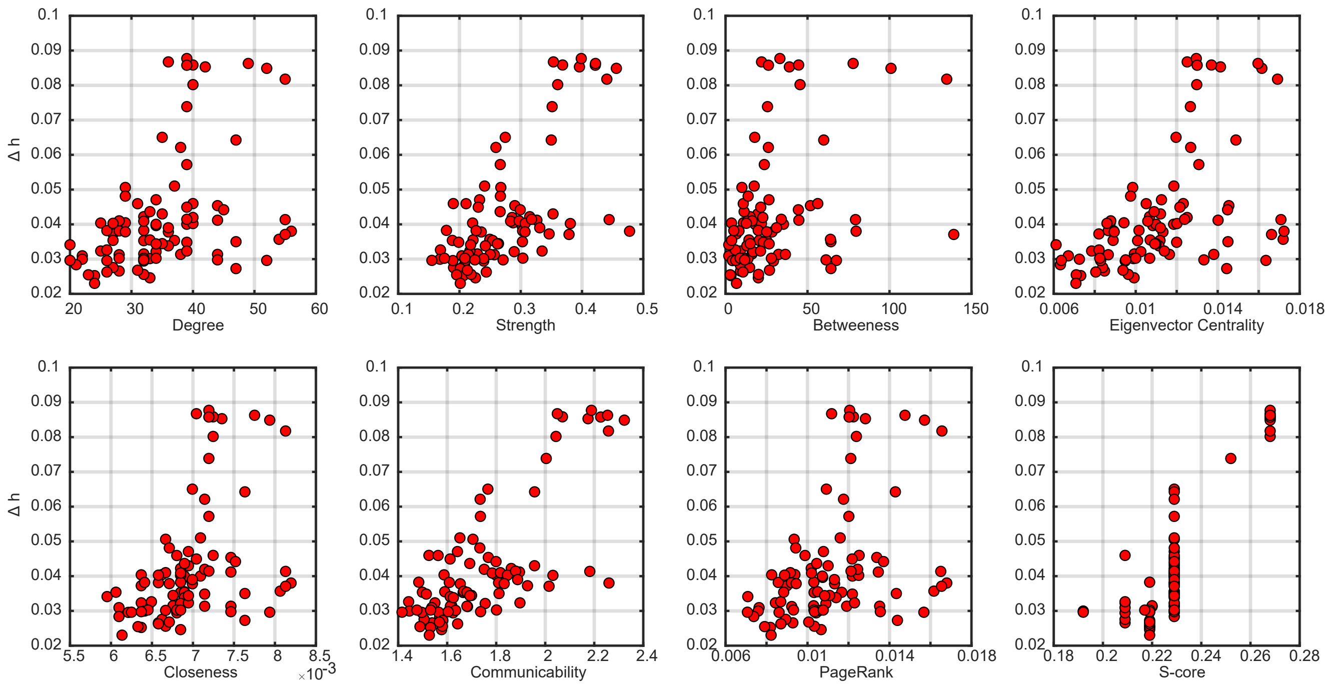

Supplementary Figure 3. Centrality measures as possible explanatory variables for Δh_n. Local connectivity strength is the best linear predictor of Δh_n among other centrality measures. Communicability also showed a good linear relationship with Δh_n, but strength has more interpretability in terms of the connectome. Circles are averages computed from 1000 independent simulations.

Supplementary Subsection 4. Receptors controls.

We controlled the role of receptor density distribution on the observed entropy changes (Δh_n) induced by 5HT2A-R activation by using alternative receptor distributions. To test the effect of the heterogeneous 5HT2A-R distribution on Δh_n, we used an uniform receptor distribution, where the receptor density for each region corresponds to the average 5HT2A-R density among all regions. To evaluate the role of the specific 5HT2A-R distribution, a randomised version of the 5HT2A-R was also used, where for each simulation the receptor expression is randomised. Finally, to evaluate the effect of activating other serotonin receptors, we used the 5HT4-R distribution, a receptor thought to play no role on the psychedelic state.

The Uniform and Random receptor distributions show almost the same complex behaviour than the 2A on average. The 5HT4-R distribution changes entropy but in a subtler manner, showing no complex dependency with the strength. On summary, the specific 5HT2A-R distribution does not impact critically on Δh_n values; rather, the average receptor density seems to capture most of the relationship with the connectivity strength. In addition, using other empirically-based distribution yields no significant change in average brain entropy, highlighting the key relationship between 5HT2A-R density and connectivity on the entropic effects induced by serotonergic psychedelic drugs.

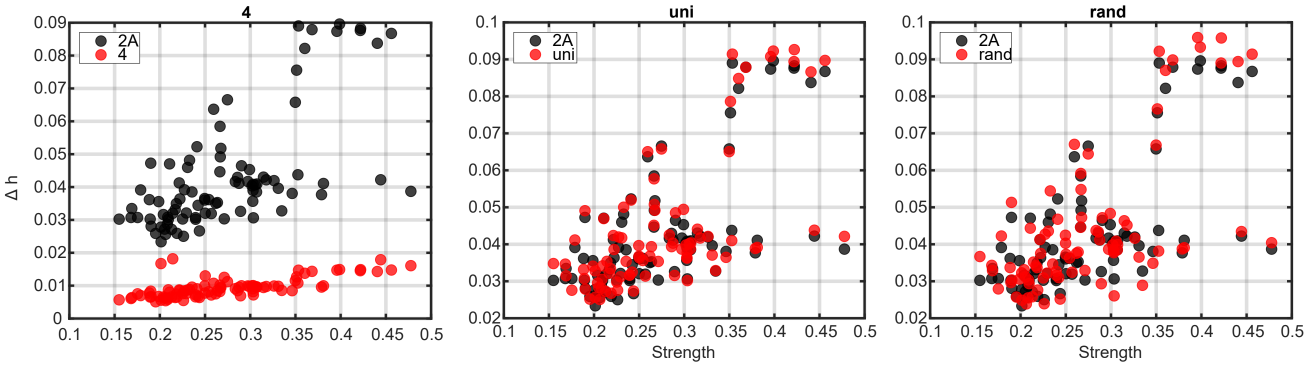

Supplementary Figure 4. Controls for receptor distribution vs Δh_n. Δh_n vs connectivity strength is plotted for 5HT2A-R, Uniform, 5HT4-R, and Random distribution of receptors (see text for details). The specific distribution of 5HT2A-R is critical both for the heterogeneity of Δh_n and for the average increase in local entropy. Circles are averages from 1000 independent simulations.

Supplementary Subsection 5. The probability distribution of excitatory firing rates can be well fitted by a Gamma distribution.

To check the goodness of fit (GOF) of Gamma distribution to the simulated excitatory firing rates of each region, we generated 10^6 simulation points for each region under both PLA and 5HT2A condition, then, a Gamma distribution was fitted and the Kolmogorov-Smirnov (K-S) distance between the firing rate distribution and 1000 random samples (same size) of the respective fitted Gamma distribution was computed. We summarise the Gamma GOF as the average K-S for each region under both conditions. To assess the significance of average GOF values, we generated an acceptance interval computing the K-S distance between 100 independent samples (same size) sampled from exactly the same Gamma distribution (re-sampled K-S). This procedure was repeated for different set of Gamma distribution parameters. If the average K-S distance of a given region falls below the maximum re-sampled K-S distance, we consider this region to be well fitted by the Gamma distribution.

Despite the decreased GOF under 5HT2A condition (compared to PLA) all regions fall within the acceptance interval under both conditions, enabling us to use the Gamma distribution parameters to estimate each region's Shannon's differential entropy.

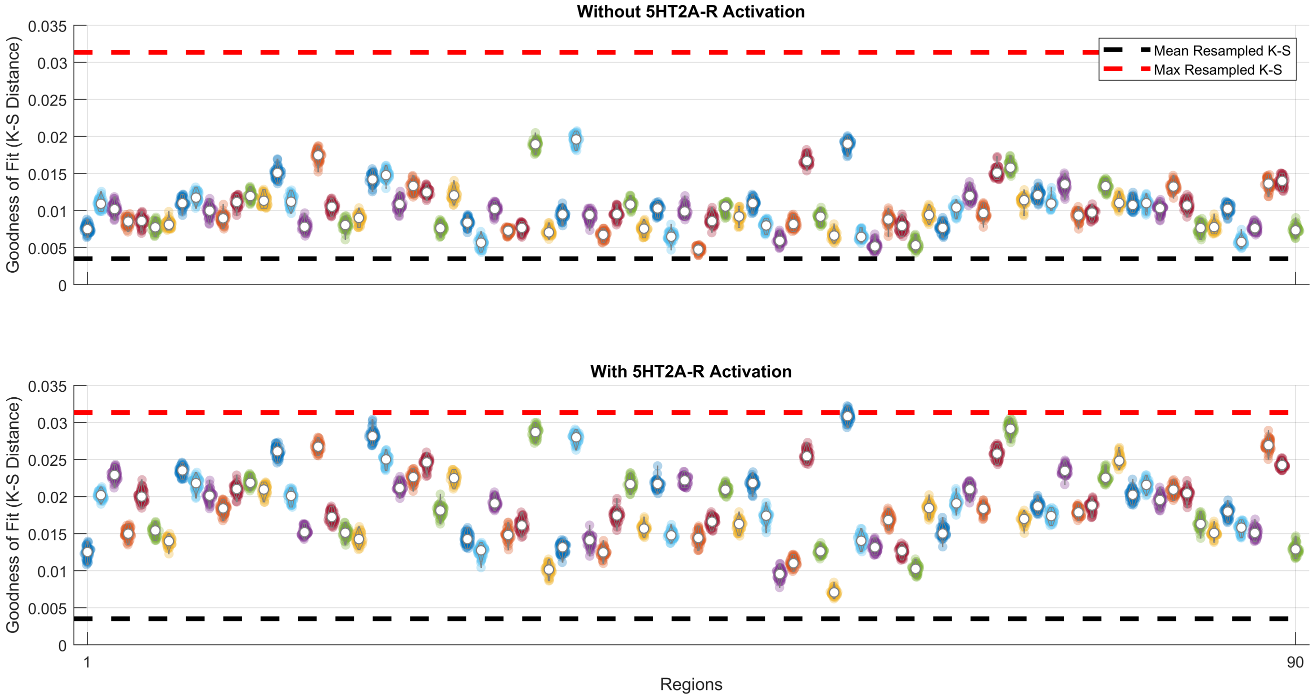

Supplementary Figure 5. Goodness of fit of Gamma distribution to firing rates distribution are within confidence intervals. Distribution of K-S distance values for PLA (top) and 5HT2A-R (bottom) condition for each region are represented by violins, where white circle denotes average K-S. Black (red) dashed line represent the average (maximum) re-sampled K-S distance (see the text for details).

Supplementary Subsection 6. Brain Regions of the Automated Anatomical Labeling.

Supplementary Table 1. AAL regions and abreviations

AAL Abbreviation	Brain Regions
Precentral	Precental gyrus
Front Sup	Superior frontal gyrus, dorsolateral
Front Sup Orb	Superior frontal gyrus, orbital part
Front Mid	Middle frontal gyrus
Front Mid Orb	Middle frontal gyrus, orbital part
Front Inf Oper	Inferior frontal gyrus, opercular part
Front Inf Tri	Inferior frontal gyrus, triangular part
Front Inf Orb	Inferior frontal gyrus, orbital part
Rolandic Oper	Rolandic operculum
Supp Motor Ar	Supplementary motor area
Olfactory	Olfactory cortex
Frontal Sup Med	Superior frontal gyrus, medial
Frontal Mid Orb	Superior frontal gyrus, medial orbital
Rectus	Gyrus rectus
Insula	Insula
Cingulum Ant	Anterior cingulate and paracingulate gyri
Cingulum Mid	Median cingulate and paracingulate gyri
Cingulum Post	Posterior cingulate gyrus
Hippocampus	Hippocampus
ParaHippocamp	Parahippocampal gyrus
Amygdala	Amygdala
Calcarine	Calcarine fissure and surrounding cortex
Cuneus	Cuneus
Lingual	Lingual gyrus
Occipital Sup	Superior occipital gyrus
Occipital Mid	Middle occipital gyrus
Occipital Inf	Inferior occipital gyrus
Fusiform	Fusiform gyrus
Postcentral	Postcentral gyrus
Parietal Sup	Superior parietal gyrus
Parietal Inf	Inferior parietal, but supramarginal and angular gyri
SupraMarginal	Supramarginal gyrus
Angular	Angular gyrus
Precuneus	Precuneus
Paracentral Lobule	Paracentral lobule
Caudate	Caudate nucleus
Putamen	Lenticular nucleus, putamen
Pallidum	Lenticular nucleus, pallidum
Thalamus	Thalamus
Heschl	Heschl gyrus
Temporal Sup	Superior temporal gyrus
Temporal Pol Sup	Temporal pole: superior temporal gyrus
Temporal Mid	Middle temporal gyrus
Temporal Pol Mid	Temporal pole: middle temporal gyrus
Temporal Inf	Inferior temporal gyrus

Supplementary Subsection 7. Anatomical and Functional Grouping of Brain Regions.

Supplementary Table 2. Anatomical Grouping

Anatomical Group	AAL Regions
Cingulate	Cingulum Ant<br>Cingulum Mid<br>Cingulum Post
Frontal	Frontal Sup<br>Front Sup Orb<br>Front Mid<br>Front Mid Orb<br>Front Inf Ope<br>Front Inf Tri<br>Front Inf Orb<br>Supp Motor Ar<br>Olfactory<br>Front Sup Med<br>Front Med Orb<br>Rectus
Limbic	Hippocampus<br>ParaHippocamp<br>Amygdala
Occipital	Calcarine<br>Cuneus<br>Lingual<br>Occipital Sup<br>Occipital Mid<br>Occipital Inf<br>Fusiform
Parietal	Parietal Sup<br>Parietal Inf<br>SupraMarginal<br>Angular<br>Precuneus<br>Paracentr Lob
Sensorimotor	Parietal Sup<br>Parietal Inf<br>SupraMarginal<br>Angular<br>Precuneus<br>Paracentr Lob
Subcortical	L Caudate<br>Putamen<br>Pallidum<br>Thalamus
Temporal	Insula<br>Heschl<br>Temporal Sup<br>Tempr Pol Sup<br>Temporal Mid<br>Tempr Pol Mid<br>Temporal Inf

Supplementary Table 3. Functional Grouping. If no hemisphere is specified, then regions from both left (L) and right (R) hemisphere are used.

Functional Group	AAL Regions
Visual (Vis)	Calcarine
	Cuneus
	Lingual
	Occipital Sup (L)
Extrastriate (ES)	Lingual (L)
	Occipital Sup
	Occipital Mid
	Occipital Inf
	Parietal Sup
Auditory (Aud)	Front Inf Ope (L)
	Front Inf Tri (L)
	Rolandic Oper
	Insula
	Cingulum Ant
	Cingulum Mid
	Amygdala
	SupraMarginal
	Putamen
	Pallidum
	Thalamus
	Heschl
	Temporal Sup
	Temporal Mid (R)
	Tempr Pol Sup (R)
Somatomotor (SM)	Heschl
	Paracentr Lob
	Parietal Sup
	Postcentral
	Cingulum Mid
	Rolandic Oper
	Precentral (R)
Excecutive Control (EC)	Precentral
	Frontal Sup
	Front Mid
	Supp Motor Ar
	Front Sup Med
	Front Med Orb
	Insula
	Cingulum Ant
	Cingulum Mid
	SupraMarginal (L)
	Precuneus
	Tempr Pol Sup
	Caudate (R)
Frontoparietal (FPN)	Front Mid
	Front Inf Ope
	Front Inf Tri
	Parietal Inf
	Angular
Default Mode (DMN)	Front Sup Med
	Cingulum Ant
	Cingulum Post
	Angular
	Precuneus
Salience (Sal)	Front Sup Orb
	Front Mid
	Front Mid Orb
	Front Inf Orb
	Supp Motor Ar
	Insula
	Cingulum Ant

Supplementary Subsection 8. Tractography.

For the sake of completeness, in the following we describe in detail the methods used by Ref.[1] to obtain the human connectome. We clarify that we did not produce these connectome data sets, instead, we used data kindly provided by Gustavo Deco.

The structural connectivity between the 90 AAL regions was obtained from averaging across 16 healthy young adults (5 females, mean SD age: 24.75 2.54). The linear registration tool from the FSL toolbox (www. fmrib.ox.ac.uk/fsl, FMRIB, Oxford) was used to coregister the EPI image to the T1-weighted structural image. The T1-weighted image was co-registered to the T1 template of ICBM152 in MNI space. The resulting transformations were concatenated and inversed and further applied to warp the AAL template from MNI space to the EPI native space, where interpolation using nearest-neighbor method ensured that the discrete labeling values were preserved. Thus the brain parcellations were conducted in each individual's native space. The structural connectivity (SC) maps were generated for each participant using the dMRI data acquired. The two data sets acquired have different phase encoding to optimize signal in difficult regions. The construction of these structural connectivity maps consisted of a three-step process. First, the regions of the whole-brain network were defined using the AAL template as used in the functional MRI data. Second, the connections between nodes in the whole-brain network (i.e., edges) were estimated using probabilistic tractography. Third, data was averaged across participants.

The FSL diffusion toolbox (Fdt) was used to carry out the various processing stages of the diffusion MRI data using the default parameters of this imaging pre-processing pipeline on all participants. Following this preprocessing, we estimated the local probability distribution of fiber direction at each voxel. The probtrackx tool in Fdt was used to provide automatic estimation of crossing fibers within each voxel.

The connectivity probability from a seed voxel i to another voxel j was defined by the proportion of fibers passing through voxel i that reach voxel j using a sampling of 5000 streamlines per voxel. This was extended from the voxel level to the region level, i.e., in an AAL parcel consisting of n voxels, 5000x n fibers were sampled. The connectivity probability P_{ij} from region i to region j is calculated as the number of sampled fibers in region i that connect the two regions divided by 5000 x n, where n is the number of voxels in region i. The SC matrix was thresholded at 0.1%, i.e., five streamlines.

For each brain region, the connectivity probability to each of the other 89 regions within the AAL was calculated. Due to the dependence of tractography on the seeding location, the probability from i to j is not necessarily equivalent to that from j to i. However, these two probabilities are highly correlated across the brain for all participants (the least Pearson $r = 0.70$, $p < 10^{-}50$). As directionality of connections cannot be determined based on diffusion MRI, the unidirectional connectivity probability P_{ij} between regions i and j was defined by averaging these two connectivity probabilities. This unidirectional connectivity was considered as a measure of the structural connectivity between the two areas, with $C_{ij} = C_{ji}$. The regional connectivity probability was calculated using in-house Perl scripts. For both phase encoding directions, 90x90 symmetric weighted networks were constructed based on the AAL90 parcellation, and normalized by the number of voxels in each AAL region; thus representing the structural connectivity network organization of the brain.

References

1. Deco, G. et al. Whole-brain multimodal neuroimaging model using serotonin receptor maps explains non-linear functional effects of LSD. Curr. Biol. 1–10, DOI: 10.1016/j.cub.2018.07.083 (2018).
2. Deco, G., Hagmann, P., Romani, G. L., Mantini, D. & Corbetta, M. How local excitation-inhibition ratio impacts the whole brain dynamics. J. Neurosci. **34**, 7886–7898, DOI: 10.1523/JNEUROSCI.5068-13.2014 (2014).
3. Tzourio-Mazoyer, N. et al. Automated anatomical labeling of activations in SPM using a macroscopic anatomical parcellation of the MNI MRI single-subject brain. NeuroImage **15**, 273–89, DOI: 10.1006/nimg.2001.0978 (2002).
4. Beckmann, C. F., DeLuca, M., Devlin, J. T. & Smith, S. M. Investigations into resting-state connectivity using independent component analysis. Philos. Transactions Royal Soc. B: Biol. Sci. **360**, 1001–1013, DOI: 10.1098/rstb.2005.1634 (2005).
5. Lee, W. & Frangou, S. Linking functional connectivity and dynamic properties of resting-state networks. Sci. Reports **7**, DOI: 10.1038/s41598-017-16789-1 (2017).
6. Oliver, I., Hlinka, J., Kopal, J. & Davidsen, J. Quantifying the Variability in Resting-State Networks. Entropy **21**, 882, DOI: 10.3390/e21090882 (2019).
7. Schartner, M. M., Carhart-Harris, R. L., Barrett, A. B., Seth, A. K. & Muthukumaraswamy, S. D. Increased spontaneous MEG signal diversity for psychoactive doses of ketamine, LSD and psilocybin. Sci. Reports **7**, 46421, DOI: 10.1038/srep46421 (2017).
8. Lebedev, A. V. et al. LSD-induced entropic brain activity predicts subsequent personality change. Hum. Brain Mapp. **37**, 3203–3213, DOI: 10.1002/hbm.23234 (2016).
9. Zuo, X. N. et al. Network centrality in the human functional connectome. Cereb. Cortex **22**, 1862–1875, DOI: 10.1093/cercor/bhr269 (2012).
10. Andreotti, J. et al. Validation of network communicability metrics for the analysis of brain structural networks. PLoS ONE **9**, 1–26, DOI: 10.1371/journal.pone.0115503 (2014).
11. Rubinov, M. & Sporns, O. Complex network measures of brain connectivity: Uses and interpretations. NeuroImage **52**, 1059–1069, DOI: 10.1016/j.neuroimage.2009.10.003 (2010).
12. Castro, S., El-Deredy, W., Battaglia, D. & Orio, P. Cortical ignition dynamics is tightly linked to the core organisation of the human connectome. PLOS Comput. Biol. **16**, 1–23, DOI: 10.1371/journal.pcbi.1007686 (2020).
